# Colonization Rate and Risk Factors of Vancomycin-Resistant Enterococci among Patients Received Hematopoietic Stem Cell Transplantation in Shiraz, Southern Iran

**Published:** 2016-11-01

**Authors:** M. Kaveh, A. Bazargani, M. Ramzi, H. Sedigh Ebrahim-Saraie, H. Heidari

**Affiliations:** 1 *Department of Bacteriology and Virology, School of Medicine, Shiraz University of Medical Sciences, Shiraz, Iran*; 2 *Hematology Research Center, Department of Hematology-Oncology and Stem Cell Transplantation, Shiraz University of Medical Sciences, Shiraz, Iran*

**Keywords:** *Enterococcus*, Vancomycin, Risk factors, Colonization, Stem cell transplant

## Abstract

**Background:**

Infections caused by antimicrobial-resistant bacteria are associated with increased mortality and health care costs. Enterococci have been recognized as a clinically important pathogen in hospitalized patients. Vancomycin-resistant enterococci (VRE) infections cause significant morbidity and mortality among patients undergoing transplantation.

**Objective:**

To identify epidemiology of VRE colonization and related risk factors among patients with hematological malignancies after hematopoietic stem cell transplantation (HSCT).

**Methods:**

This cross-sectional study was performed on 42 patients who underwent bone-marrow transplantation between July 2013 and March 2014. A stool sample was taken from each patient 3–5 days after transplantation and cultured on appropriate media. Suspected colonies of enterococci were detected to species level by their culture characteristics, biochemical reactions and molecular features. VRE were confirmed via phenotypic and genotypic methods.

**Results:**

VRE were detected in 14 (33%) of studied samples. 10 (71%) of the detected VRE isolates were identified as high level vancomycin-resistant *E. faecium *with minimum inhibitory concentration (MIC) of ≥256 μg/mL of vancomycin; 3 isolates were *E. galinarum *and 1 was *E. casseliflavus *with an MIC of 8–16 μg/mL. VanA was dominant phenotype and all VRE isolates with high-level of vancomycin resistance had *vanA* gene. VRE isolation was mostly observed in patients with acute lymphoblastic leukemia (ALL) than other diseases. Moreover, antibiotic prophylaxis and hospitalization were independent risk factors for acquisition of VRE after transplantation.

**Conclusion:**

We found high level of vancomycin-resistance in *E. faecium *isolates obtained from HSCT patients*.* The vancomycin-resistant isolates of *E.* *faecium* had *vanA* and/or simultaneously *vanB* genes.

## INTRODUCTION

Emergence of antibiotics resistance and selection of the drug of choice for the treatment of nosocomial infections are among most important global health concerns [1]. Vancomycin-resistant enterococci (VRE) can be considered as one of the main challenges in this issue. In addition, VRE commonly show resistance toward many antibiotics in addition to vancomycin [2]. 

Both microbial and host factors can contribute to enterococcal nosocomial infections; it seems that increased density of gastrointestinal tract colonization by enterococci is one of the most important factors that promote these infections [3]. Nowadays, increasing rate of VRE colonization has been reported among patients who had prolonged hospitalization, especially in organ transplant wards [4, 5].

VRE infections in many countries have been associated with high morbidity and mortality rates, particularly among immuno-compromised patients [6, 7]. Resistance to vancomycin is mediated by *van* gene cluster, which are carried on transposable elements [4].

Bone marrow and stem cell transplant patients are at a higher risk of being colonized and infected with antimicrobial-resistant pathogens, particularly with VRE [3]. VRE infections are one the commonest bacterial threat among patients receiving hematopoietic stem cell transplant. Studies over the last decade have documented remarkable increase in the rates of early VRE bacteremia and mortality after hematopoietic stem cell transplant (HSCT) ranging from 3.6% to 22% and 0.04% to 85%, respectively [8]. VRE infections have been associated with very high mortality among HSCT recipients and identifying the risk factors of VRE colonization can be critical in management and reducing adverse consequence of VRE infections [9, 10].

Therefore, in this study we aimed at screening HSCT recipients for VRE stool colonization and identifying the related risk factors for patients who are at high risk of VRE bloodstream infection in the early post-transplantation period.

## PATIENTS AND METHODS

Study Design and Bacterial Isolates

This cross-sectional study was conducted between July 2013 and March 2014 on 42 recipients of bone marrow transplant who referred to Nemazee hospital, one of the most important transplant centers in Iran. The adult Bone Marrow and Stem Cell Transplant Unit in Shiraz is a 15-bed ward where patients are isolated from other hospitalized patients. One stool sample for VRE surveillance was collected from each patient 3–5 days after receiving a HSCT. Bile esculin azide agar (Quelab, Canada) was used for primary detection of *Enterococcus* isolates from specimens kept at 45 °C. Gram-positive cocci arranged in pairs or chains with black colonies on bile esculin azide agar were taken for further identification to species level according to standard microbiological tests (including PYRase, and arginine and carbohydrates fermentation). 

To assess the related risk factors in VRE colonization including blood culture results, medical information for each patient stored in the database of Bone Marrow Transplant Center was analyzed. This study was in accordance with the declaration of Helsinki and approved by the Ethics Committee of Shiraz University of Medical Sciences (EC-92-6600). An informed written consent was taken from all participants.

Antimicrobial Susceptibility Testing 

Antimicrobial susceptibility pattern was determined by disc diffusion method against penicillin (10 µg), synercid (15 µg), teicoplanin (30 µg), ampicillin (10 µg), gentamicin (120 µg), rifampin (5 µg), levofloxacin (5 µg), erythromycin (15 µg), tetracycline (30 µg), chloramphenicol (30 µg), nitrofurantoin, (300 µg), fosfomycin (200 µg), and linezolide (30 µg) (MAST Diagnostics, UK) on Müeller-Hinton agar (Merck, Germany) according to recommendations of the Clinical and Laboratory Standard Institute (CLSI) [11]. The vancomycin minimal inhibitory concentration (MIC) was evaluated by the E-test method (Liofilchem MIC Test Strip, Italy) and VRE isolates with MIC of ≥32 µg/mL was considered for further genotyping by polymerase chain reaction (PCR).

Molecular Analysis

PCR was performed to determine the glycopeptides resistance genotypes and species confirmation. Bacterial DNA was extracted by the boiling method at 100 °C for 10 min. The PCR analyses were performed in a DNA Thermal Cycler 5530 (Ependrof master, Germany) for detecting the presence of *vanA*, *B*, *C*1 and *C*2/*C*3 genes among VRE isolates and internal genes for confirmation of isolates at species level. Previously designed primers (Cinna Gen Co, Iran) were used for amplification of *vanA* (734-bp), *vanB* (297-bp) [12], *vanC1* (531-bp), and *vanC*2/*C*3 (673-bp) [13], *E. faecalis *(941-bp), and *E. faecium *(658-bp) [14] (Table 1). Reference strains ATCC 51599 (*E. faecium*), ATCC 51299 (*E. faecalis*), ATCC 49573 (*E. gallinarum*), and ATCC 25788 (*E. casseliflavus*) were used as positive controls for detection of the desired genes in PCR method. Moreover, *E. faecalis* ATCC 29212 was used as a negative control for vancomycin resistance genes. 

**Table 1:** PCR primers used in the present study

Primer	Size of PCR product (bp)	Primer pair sequences (left to right 5’–3’)
*vanA* Forward	734	AATACTGTTTGGGGGTTGCTC
*vanA* Reverse		CTTTTTCCGGCTCGACTTCCT
*VanB* Forward	297	CATCGCCGTCCCCGAATTTCAAA
*VanB* Reverse		GATGCGGAAGATACCGTGGCT
*vanC1* Forward	531	TTGACCCGCTGAAATATGAAGTAA
*vanC1* Reverse		TAGAACCGTAAGCAAAAGCAGTCG
*vanC2*/3 Forward	673	GCATGGCAAATACGGGGAAGAT
*vanC2/3* Reverse		CATGGCAGGATAGCGGGAGTGA
*E. faecalis* Forward	941	ATCAAGTACAGTTAGTCTTTATTAG
*E. faecalis* Reverse		ACGATTCAAAGCTAACTGAATCAGT
*E.* *faecium* Forward	658	TTGAGGCAGACCAGATTGACG
*E.* *faecium* Reverse		TATGACAGCGACTCCGATTCC

Statistical Analysis 

Data were analyzed with SPSS^®^ for Windows^®^ ver 21 (IBM Corp, USA). Qualitative variables were compared with χ^2^ test. Odds ratio and 95% confidence intervals were calculated by logistic regression analysis.

## RESULTS

Of 42 patients studied, 22 had received autologous bone marrow transplants and 22 had received allogeneic transplants. Two patients had had both types of transplantations. Of 42 patients, 27 (64%) were male. Fourteen (33%) patients were found colonized with VRE, 19 (45%) were colonized with vancomycin-sensitive enterococci (VSE), and 9 (21%) showed negative growth for enterococci. Of the 19 VSE isolates, 11 were *E. faecalis*, 5 *E. faecium*, 2 *E. gallinarum*, and one isolate was* E. casseliflavus*. The commonest isolates of VRE were *E. faecium* found in 10 (71%) with MIC of ≥256 µg/mL, *E. gallinarum* in 3 (21%) patients with MIC of 12–16 µg/mL, and *E. Casseliflavus* isolated from one (7%) with MIC of 8 µg/mL.

**Figure 1:**
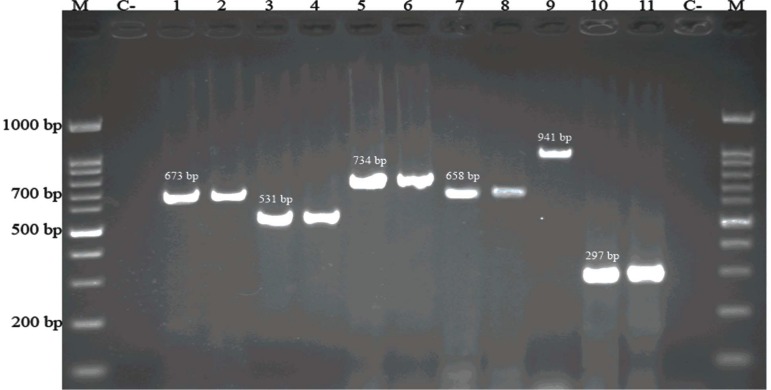
Representative image of agarose gel electrophoresis for studied genes by the PCR assay. M: 100-bp DNA ladder; C: negative control; lanes 1 to 11 for each gene, a positive control and a positive sample is placed. Lane 1-2 *vanC*2/*C*3 (673-bp), lane 3-4 *vanC1,* lane 5-6* vanA* (734-bp), lane 7-8 *E. faecium *conserved gene (658-bp), lane 9 *E. faecalis *(941-bp), lane 10-11* vanB* (297-bp

All *E.* *faecium* isolates had *vanA* genotype; 3 of which also carried *vanB* gene.* E. gallinarum* isolates had *vanC1* gene. The only *E. casseliflavus *isolate carried *vanC2*/*3* gene (Fig 1).

Results of antimicrobial susceptibility tests revealed that all VRE isolates were susceptible to linezolid and fosfomycine with the least resistance to synercid. All vancomycin-resistant *E.* *faecium* were also resistant to ampicillin, erythromycin, penicillin, levofloxacin, and gentamicin. Complete results of antibiotic susceptibility tests and genotypes associated antibiotic resistance among VRE isolates are shown in Tables 2 and 3, respectively. 

**Table 2:** Antibiotic susceptibility patterns of vancomycin-resistant enterococci isolates

Susceptibility Antibiotics	Resistant n (%)	Intermediate n (%)	Sensitive n (%)
Ampicillin	12 (86)	—	2 (14)
Penicillin	13 (93)	—	1 (7)
Teicoplanin	7 (50)	—	7 (50)
Nitrofurantoin	8 (57)	—	6 (43)
Rifampin	12 (86)	—	2 (14)
Erythromycin	14 (100)	—	—
Levofloxacin	14 (100)	—	—
Chloramphenicol	8 (57)	—	6 (43)
Linezolid	—	—	14 (100)
Synercid	2 (14)	1 (7)	11 (79)
Tetracycline	10 (71)	—	4 (29)
Gentamicin	14 (100)	—	—
Fosfomycin	1 (7)	—	13 (93)

**Table 3:** Antibiotic resistance patterns of vancomycin-resistant enterococci isolates according to genotype

Antibiotic resistance pattern	Number of isolates contain, *vanA *or *vanB*	Number of isolates contain, *vanC*
Ampicillin	10	2
Penicillin	10	3
Teicoplanin	7	0
Nitrofurantoin	7	1
Rifampin	9	3
Erythromycin	10	4
Levofloxacin	10	4
Chloramphenicol	8	0
Linezolid	0	0
Synercid	3	0
Tetracycline	8	2
Gentamicin	10	4
Fosfomycin	1	0

Isolation of VREs was mostly in patients with acute lymphoblastic leukemia (ALL) compared to other groups (Table 4). History of antibiotic use during the last three months (OR 6.60, 95% CI: 1.60–27.24) was found as an independent risk factor for VRE colonization in transplanted patients. Previous history of hospitalization (OR 6.10, 95% CI: 0.69–54.64), and ICU stay (OR 3.67, 95% CI: 0.92–14.62), though not statistically significant, were also associated with risk of VRE acquisition (Table 5). No *Enterococcus* spp. was isolated from the blood specimens taken from the studied transplant recipients.

**Table 4:** Frequency of underlying disease and rate of entrococci colonization

Underlying disease	VRE n (%)	VSE n (%)	Not colonized n (%)
ALL (acute lymphoblastic leukemia)	6 (43)	2 (11)	0
AML (acute myeloid leukemia)	0	6 (32)	1 (11)
HL (Hodgkin’s lymphoma)	1 (7)	2 (11)	2 (22)
NHL (Non-Hodgkin’s lymphoma)	3 (21)	2 (11)	0
Multiple myeloma	2 (14)	6 (32)	4 (44)
Immunodeficiency syndrome	1 (7)	1 (5)	0
SCID (Severe combined immunodeficiency)	1 (7)	0	1 (11)
Fanconi anemia	0	0	1 (11)
Total	14 (33)	19 (45)	9 (21)

**Table 5:** Risk factors for colonization of vancomycin-resistant enterococci after transplantation for bone marrow transplant recipients

Variable	VRE patients (n =14)	NO VRE patients (n =28)	Odd Ratio(95% CI)
Age (yrs)			
0–25	4 (29)	8 (29)	Reference
25–50	6(43)	13 (46)	0.92 (0.20–4.31)
>50	4 (29)	7 (25)	1.14 (0.21–6.37)
Male sex	9 (64)	18 (64)	1.00 (0.26–3.82)
Surgery	3 (21)	9 (32)	0.58 (0.13–2.59)
Gastrointestinal bleed	1 (7)	2 (7)	1.00 (0.08–12.07)
Gastrointestinal disease	2 (14)	5 (18)	0.77 (0.13–4.56)
Albumin (g/L)			
2–2.5	2 (14)	0	
2.5–3	1 (7)	2 (7)	1.33 (0.09–20.71)
3–3.5	1 (7)	5 (18)	0.53 (0.043–6.66)
3.5–4	7 (50)	12 (43)	1.44 (0.29–7.21)
>4	3 (21)	9 (32)	Reference
Antibacterial treatment			
Carbapenem	6 (43)	12 (43)	1.00 (.27–3.66)
Vancomycin	7 (50)	7 (25)	3.00(0.776–11.60)
Third-generation cephalosporins	11 (79)	21 (75)	1.47 (0.32–6.69)
Metronidazole	1 (7)	1 (4)	1.47 (0.32–6.69)
Antifungal drugs	6 (43)	6 (21)	2.75 (0.68–11.05)
Previous antibiotic use (past 3 months)	9 (64)	6 (21)	6.60 (1.60–27.24)
Diabetes	5 (36)	7 (25)	1.67 (0.42–6.68)
Previous hospitalization (one year ago)	13 (93)	13 (68)	6.16 (0.69–54.64)
Immunosuppressive drugs before transplantation	10 (71)	14 (50)	2.50 (0.63–9.90)
Type of transplant			
Allogeneic	9 (64)	13 (68)	1.33 (0.37–4.85)
Autologous	5(36)	17 (61)	Reference
Hospitalized in BMT ward before transplantation (day)			
0–5	1 (7)	1 (4)	0.25 (0.01–4.73)
5–10	5 (36)	20 (72)	1.14 (0.06–21.87)
>10	8 (57)	7 (25)	Reference
Admission to the ICU	8 (57)	6 (21)	3.67 (0.92–14.62)
Duration of disease (yrs)			
0–1	8 (57)	12 (43)	2.05 (0.43–9.78)
1–2	3 (21)	6 (21)	2.00 (0.29–13.74)
>2	3 (21)	10 (36)	Reference

BMT: Bone marrow transplantation; ICU: Intensive care unit; VRE: vancomycin-resistant enterococci

* Female gender and negative responses have been as the basis considered in the calculations

## DISCUSSION

To best of our knowledge, there was no published information on the frequency of VRE colonization and associated risk factors in bone-marrow transplant recipients from Iran and the present study is the first of its kind from our region.

In US hospitals, enterococci are the second most common organism recovered from skin, soft-tissue and catheter associated with bloodstream infections [3]. Nosocomial infections caused by VRE are major concern at many hospitals around the world including Iran. VRE is now considered one of the most common causes of bacteremia in critically ill and neutropenic patients with cancer [6, 15, 16]. Because of multi-drug resistance nature of VRE infections, early treatment options are limited; additionally, VRE bacteremia is associated with a high mortality rate [8]. In our results, the VRE colonization was observed in 33% of HSCTs, which reflects the importance of enterococcal infections in these patients. Most of our studied patients were neutropenic (data not shown), immunosuppressed, and had history of hospitalization, which may explain higher VRE colonization rate in our study compared to reports from other parts of the world. A recent study by Jan Vydra, *et al*, on patients receiving allogeneic hematopoietic stem cells transplant in the USA indicated that 23% of patients were found colonized with VRE throughout the study period [17]. In another study in pediatric stem cell transplant patients from the USA, 24.6% of patients had positive stool culture for VRE [7]. There is no similar study in Iran from patients receiving hematopoietic stem cells, but a study among children with ALL at two referral centers of Tehran, Iran reported a 25% rate of VRE colonization [18].

Since the increasing use of broad-spectrum antibiotics in the 1990s that followed by an increase in VRE infections, *E. faecalis* has been known as a common cause of enterococcal nosocomial infections [3]. However, recent reports indicated that the organism has been replaced by *E. faecium* [3,12]. This change has serious clinical implications, infections caused by *E*. *faecium* are far more difficult to treat because of its intrinsically resistance to most of the common antibiotics [3,12]. 

The dominant species in the present study was *E. faecium* with *vanA* gene. In many parts of the world such as the USA, France, Italy, Argentina, and South Korea, the results of molecular study and phenotypes of vancomycin resistance show an increase in the prevalence of *E. faecium* with *vanA* gene [7, 20-23]. In another study conducted in Turkey, *E. faecium* with *vanB *gene was the dominant species [24].

Colonization and infection with VRE are affected by a variety of risk factors, *e.g.*, hospitalization time, the underlying disease, transplantation, and use of vancomycin or third-generation cephalosporins [25-28]. All patients in our study received ceftazidime immediately after the transplantation. Moreover, some of the patients additionally received vancomycin or carbapenem. Furthermore, 38 patients had hematologic malignancies and four suffered from a genetic defect. Previous use of antibiotics in the last three months prior to transplantation (p<0.009) was significantly associated with colonization of VRE and patients with hospitalization more than 10 days prior to transplantation showed higher tendency for VRE acquisition. 

More than 85% of the tested VRE isolates were resistance to six antibiotics in common use—penicillin, ampicillin, rifampin, erythromycin, levofloxacin, and gentamicin. These results were similar to those obtained by Talebi, *et al*, from Iran and Bourdon, *et al*, from France [20, 29]. On the other hand, most of our VRE isolates were sensitive to linezolid, fosfomycin, and synercid. Our results, like many other studies where resistance of VRE isolates to linezolid was reported in low frequencies, suggested linezolid as the first line of treatment for VRE infections [20, 30-32]. In some parts of the world, including Iran, due to difficulties in the preparation of some drugs, such as linezolid, there should be alternatives; we showed fosfomycin can be used effectively as a substitute for linezolid.

The present study had some limitations. The first was lack of a molecular typing method to determine the clinical relevance of the isolated enterococci. Second, our specimens were only obtained after transplantation, therefore, we could not comment on the time of colonization.

## References

[1] Spellberg B, Guidos R, Gilbert D (2008). The epidemic of antibiotic-resistant infections: a call to action for the medical community from the Infectious Diseases Society of America. Clin Infect Dis.

[2] Murray BE (2000). Vancomycin-resistant enterococcal infections. N Engl J Med.

[3] Arias CA, Murray BE (2012). The rise of the Enterococcus: beyond vancomycin resistance. Nat Rev Microbiol.

[4] Cetinkaya Y, Falk P, Mayhall CG (2000). Vancomycin-resistant enterococci. Clin Microbiol Rev.

[5] Freitas MC, Pacheco-Silva A, Barbosa D (2006). Prevalence of vancomycin-resistant Enterococcus fecal colonization among kidney transplant patients. BMC Infect Dis.

[6] Armeanu E, Bonten MJ (2005). Control of vancomycin-resistant enterococci: one size fits all?. Clin Infect Dis.

[7] Tsiatis AC, Manes B, Calder C (2004). Incidence and clinical complications of vancomycin-resistant enterococcus in pediatric stem cell transplant patients. Bone Marrow Transplant.

[8] Kamboj M, Chung D, Seo SK (2010). The changing epidemiology of vancomycin-resistant Enterococcus (VRE) bacteremia in allogeneic hematopoietic stem cell transplant (HSCT) recipients. Biol Blood Marrow Transplant.

[9] Weinstock DM, Conlon M, Iovino C (2007). Colonization, bloodstream infection, and mortality caused by vancomycin-resistant enterococcus early after allogeneic hematopoietic stem cell transplant. Biol Blood Marrow Transplant.

[10] Hendrix CW, Hammond JM, Swoboda SM (2001). Surveillance strategies and impact of vancomycin-resistant enterococcal colonization and infection in critically ill patients. Ann Surg.

[11] CLSI (2014). Performance Standards for Antimicrobial Susceptibility Testing; 24th Informational Supplement. M100-S24.

[12] Li S, Zhang Z, Mi ZH (2007). Vancomycin-resistant enterococci in a Chinese hospital. Curr Microbiol.

[13] Khan SA, Nawaz MS, Khan AA (2005). Molecular characterization of multidrug-resistant Enterococcus spp from poultry and dairy farms: detection of virulence and vancomycin resistance gene markers by PCR. Mol Cell Probes.

[14] Kariyama R, Mitsuhata R, Chow JW (2000). Simple and reliable multiplex PCR assay for surveillance isolates of vancomycin-resistant enterococci. J Clin Microbiol.

[15] Honarm H, Falah Ghavidel M, Nikokar I (2012). Evaluation of a PCR assay to detect enterococcus faecalis in blood and determine glycopeptides resistance genes: van A and van B. Iran J Med Sci.

[16] Assadian O, Askarian M, Stadler M (2007). Prevalence of vancomycin-resistant enterococci colonization and its risk factors in chronic hemodialysis patients in Shiraz, Iran. BMC Infect Dis.

[17] Vydra J, Shanley RM, George I (2012). Enterococcal bacteremia is associated with increased risk of mortality in recipients of allogeneic hematopoietic stem cell transplantation. Clin Infect Dis.

[18] Nateghian A, Robinson JL, Arjmandi K (2011). Epidemiology of vancomycin-resistant enterococci in children with acute lymphoblastic leukemia at two referral centers in Tehran, Iran: a descriptive study. Int J Infect Dis.

[19] Sydnor ER, Perl TM (2011). Hospital epidemiology and infection control in acute-care settings. Clin Microbiol Rev.

[20] Bourdon N, Fines-Guyon M, Thiolet JM (2011). Changing trends in vancomycin-resistant enterococci in French hospitals, 2001-08. J Antimicrob Chemother.

[21] Fallico L, Boldrin C, Grossato A (2011). Molecular epidemiology of Enterococcus faecium isolates from an Italian hospital. Infection.

[22] Corso AC, Gagetti PS, Rodriguez MM (2007). Molecular epidemiology of vancomycin-resistant Enterococcus faecium in Argentina. Int J Infect Dis.

[23] Yoo SJ, Sung H, Cho YU (2006). Role of horizontal transfer of the transposon Tn1546 in the nosocomial spread of vanA vancomycin-resistant enterococci at a tertiary care hospital in Korea. Infect Control Hosp Epidemiol.

[24] Yilmaz NO, Agus N, Yurtsever SG (2009). Prevalence and risk factors associated with vancomycin resistant enterococci colonization. R Ci méd biol Salvador.

[25] Gold HS (2001). Vancomycin-resistant enterococci: mechanisms and clinical observations. Clin Infect Dis.

[26] McNeil SA, Malani PN, Chenoweth CE (2006). Vancomycin-resistant enterococcal colonization and infection in liver transplant candidates and recipients: a prospective surveillance study. Clin Infect Dis.

[27] Almyroudis NG, Lesse AJ, Hahn T (2011). Molecular epidemiology and risk factors for colonization by vancomycin-resistant Enterococcus in patients with hematologic malignancies. Infect Control Hosp Epidemiol.

[28] Askarian M, Afkhamzadeh R, Monabbati A (2008). Risk factors for rectal colonization with vancomycin-resistant enterococci in Shiraz, Iran. Int J Infect Dis.

[29] Talebi M, Pourshafie MR, Oskouii M (2008). Molecular analysis of vanHAX element in vancomycin resistant enterococci isolated from hospitalized patients in Tehran. Iran Biomed J.

[30] Shokoohizadeh L, Mobarez AM, Zali MR (2013). High frequency distribution of heterogeneous vancomycin resistant Enterococcous faecium (VREfm) in Iranian hospitals. Diagn Pathol.

[31] Nateghian A, Ghasemi Ahari SM, Lahouti Harahdashti A (2014). Prevalence of Vancomycin-resistant Enterococci Colonization, and Susceptibility to linezolid in Pediatric Intensive Care Units of a Referral Pediatric Center in Tehran, Iran. Arch Pediatr Infect Dis.

[32] Wang L, He Y, Xia Y (2014). Investigation of mechanism and molecular epidemiology of linezolid-resistant Enterococcus faecalis in China. Infect Genet Evol.

